# Migration Status and HIV Pre-exposure Prophylaxis (PrEP) Uptake Among Gay and Bisexual Men and Non-binary People in Australia: Results of a National Cross-Sectional Survey

**DOI:** 10.1007/s10461-026-05070-0

**Published:** 2026-02-12

**Authors:** Simin Yu, James MacGibbon, Benjamin R. Bavinton, Sarah K. Calabrese, Dean A. Murphy, Jeanne Ellard, John de Wit, Timothy R. Broady, Martin Holt

**Affiliations:** 1 Centre for Social Research in Health, University of New South Wales, Sydney, NSW 2052 Australia; 2 Kirby Institute, UNSW Sydney, Sydney, Australia; 3 Department of Psychological and Brain Sciences, George Washington University, Washington, DC USA; 4 Australian Research Centre in Sex, Health and Society, La Trobe University, Bundoora, Australia; 5 School of Population Health, UNSW Sydney, Sydney, Australia; 6 School of Translational Medicine, Monash University, Melbourne, Australia; 7 Department of Interdisciplinary Social Science, Utrecht University, Utrecht, Netherlands

**Keywords:** Pre-exposure prophylaxis (PrEP), Migrant, Gay and bisexual men, Non-binary people, HIV prevention, Uptake, Willingness

## Abstract

Increasing HIV prevention coverage among migrant gay and bisexual men (GBM) and non-binary people in Australia has become a priority, as recently arrived migrants are at higher risk of HIV acquisition than longer-term Australian residents. We compared GBM and non-binary participants who were willing to use but had never used pre-exposure prophylaxis (PrEP) before with current PrEP users, and explored how migration status was associated with PrEP uptake. A national, online cross-sectional survey of GBM and non-binary people was conducted in June–July 2023. Migration status was measured by region of birth and length of residency in Australia. Logistic regression was used to examine migration status, along with sociodemographic, behavioural, and health characteristics, as predictors of PrEP status. Of 2046 respondents, 1907 (93.2%) were HIV-negative/untested with 947 (80.6%) currently using PrEP and another 228 (19.4%) willing to use (but never having used) PrEP. Among PrEP users and willing participants (*n* = 1175), 28.3% were overseas-born including 4.1% who had recently arrived in Australia (< 2 years). Among recently-arrived willing non-PrEP-users, 87.5% were not covered by Medicare (Australia’s national health insurance scheme). Compared to current PrEP users, willing non-PrEP-users were younger, and more likely to be recently-arrived migrants, identify as bisexual/queer/other, and lived in regional and remote areas. They were also less likely to be in paid employment, know other PrEP users, or report recent STI diagnosis. Free or low-cost access schemes, supported by translators, publicised through educational institutions and relevant social networks may help promote PrEP uptake among recently-arrived GBM and non-binary people.

## Resumen

Comparamos a los participantes HGB y no binarios que estaban dispuestos a usar la profilaxis preexposición (PrEP) pero nunca la habían usado, con los usuarios actuales de PrEP, y exploramos cómo su estatus migratorio se asociaba con la adopción de PrEP. En comparación con los usuarios actuales de PrEP, los participantes que estaban dispuestos a usar PrEP, pero nunca la habían usado eran más jóvenes y tenían mayor probabilidad de ser migrantes recién llegados, identificarse como bisexual/queer/otros,, y residir en zonas regionales y remotas en lugar de en áreas metropolitanas interiores. Los participantes dispuestos a usar PrEP tenían menos probabilidad de tener un empleo remunerado, conocer a otros usuarios de PrEP, haber recibido un diagnóstico de una infección de transmisión sexual en el último año. Los programas de acceso gratuito o de bajo costo, con el apoyo de traductores y difundidos a través de instituciones educativas y redes sociales relevantes, pueden ayudar a promover la adopción de PrEP entre las personas HGB y no binarias recién llegadas.

## Introduction

 In Australia’s HIV epidemic, the majority of new cases continue to be diagnosed among gay and bisexual men (GBM). Over the last decade, annual HIV diagnoses in Australia have fallen, particularly among Australian-born GBM [1]. However, this decline has been less evident among men born overseas, particularly those born in Asia, Latin America, and the Caribbean [1]. Between 2014 and 2023, annual HIV diagnoses among Australian-born men attributed to male-to-male sex declined by 64%, diagnoses among men born outside Asia decreased by 32%, while notifications among Asian-born men fluctuated but remained high [1]. These trends explain why understanding the HIV prevention needs of migrant GBM has become an essential part of Australia’s efforts to end the HIV epidemic [1–3].

Pre-exposure prophylaxis (PrEP) is highly effective for HIV prevention, with the regular use of oral antiretroviral drugs offering up to 99% protection against HIV infection [4]. The decline in HIV diagnoses in Australia has been linked to the successful rollout of PrEP [5, 6]. National behavioural surveillance data show that PrEP coverage, the proportion of non-HIV-positive participants who were both aware of, suitable for PrEP (based on behaviour and risk of HIV acquisition) and were currently using it, increased from 0.9% in 2014 to 66.8% in 2023 [7]. By the end of 2023, a total of 74,597 individuals had accessed PrEP through the Pharmaceutical Benefits Scheme (PBS, Australia’s government-subsidised medicines program) at least once, with 45,244 people receiving PrEP in the previous year [8]. However, despite these achievements, there are recognised disparities in HIV prevention coverage among GBM in Australia, particularly among overseas-born GBM [3, 7, 9].

Previous research has identified factors influencing PrEP uptake among GBM, including awareness and knowledge [10], stigma [10, 11], coverage under the national health insurance scheme (known as Medicare) [12, 13], and behaviours such as having multiple male partners, condomless sex, group sex or substance use [12, 13]. In Australia, PrEP is primarily accessed via the PBS, which requires Medicare eligibility through citizenship, permanent residency or a reciprocal healthcare agreement with another country [14]. Migrant GBM in Australia, especially those who have recently arrived, face intersecting barriers to PrEP access due to systemic and socio-cultural factors, such as lack of Medicare coverage, language barriers, and stigma about sexuality or HIV [2, 15–18]. Quantitative data on PrEP use by migrant GBM in Australia remain limited, particularly in comparison to Australian-born GBM, leaving it unclear how migration status impacts PrEP uptake in Australia. It is also important to note that a significant gap often exists between individuals’ willingness to use PrEP and actual uptake, due to barriers such as low literacy about the health system, interpersonal factors (e.g. stigma and the influence of partners and peers), and structural barriers (e.g. cost or lack of insurance) [19, 20]. To identify factors that limit uptake and provide insights for targeted interventions, it is useful to identify GBM who are willing to use PrEP but have never used it before and consider how they may differ from with those who have initiated and are currently using PrEP.

Our research aimed to (1) compare participants who were willing to use PrEP but had never used it before with current PrEP users, and (2) assess the associations between migration status and current PrEP use, as well as other factors associated with current PrEP use.

## Methods

### Participants and Procedures

Data were collected via a national online cross-sectional survey of GBM and non-binary people conducted in June-July 2023 using Qualtrics online survey software (Provo, UT). This was part of the PrEPARE Project, a repeated cross-sectional study of attitudes to different HIV prevention methods. The study was approved by the ethics committee of UNSW Sydney (HC230024) and reviewed by the community organisation ACON (D202301). The study design and methods have been described before [13, 21]. The survey was promoted via paid advertisements on Facebook, Instagram, and Grindr (the dating/hook up app). Potential participants were directed to the Qualtrics survey platform where they were required to read the study information and indicate their consent before commencing the survey. The participant information and survey were available in English, simplified Chinese, and Thai. Participants were eligible if they were 16 years of age or older, did not identify as female, did not identify as heterosexual, and lived in Australia at the time of the survey. Thus, adolescent and adult gay, bisexual and queer men (cisgender or transgender) and non-binary people residing in Australia were the primary study population.

## Measures

The questionnaire measures have been described before [13, 22, 23]. A range of variables were included in this analysis, including demographic variables; self-reported HIV status; recent sexual behaviours, including number of male partners, condomless anal intercourse with casual (CAIC) and regular partners (CAIR); recent STI diagnoses; and substance use. Additional key variables for this analysis included migration status, willingness to use PrEP, current PrEP use, and preferences for different ways of using PrEP.

Migration status was measured using two questions: country of birth and length of residency in Australia [3, 24]. In brief, participants born in Australia were categorized as Australian-born; participants born in Canada, Ireland, the UK, the USA and Aotearoa New Zealand were categorized as born in high-income English-speaking countries; participants who were born in other countries and had lived in Australia for two years or more were categorized as non-recently-arrived migrants; and participants who were born in other countries and had lived in Australia less than two years were categorized as recently-arrived migrants [3, 24].

Participants were asked if they had ever used PrEP. If they answered “No”, they were asked about their awareness of PrEP and willingness to use PrEP. Willingness was measured using a 7-item scale (Cronbach’s alpha = 0.77), including items such as “I would be willing to take PrEP to prevent getting HIV” and “I would be willing to pay for PrEP” [25]. All items were scored from ‘strongly disagree’ (1) to ‘strongly agree’ (5). Using an established methodology, participants with an average score of ≥ 4 on the 7-item scale were categorized as willing to use PrEP [25].

Current PrEP use was measured by the question “Are you currently taking PrEP?” Response options included the way participants were taking PrEP. Responses were recoded as using PrEP daily, on-demand use (‘2-1-1’), periodic use (using it daily for a limited period of time), taking PrEP another way, or not currently using PrEP.

### Statistical Analysis

Frequencies and proportions are reported for all variables. Chi-square, Kruskal-Wallis, and Fisher’s exact tests were used for bivariable analyses. We used logistic regression to compare participants who were willing to use PrEP but had never used PrEP to current PrEP users. Covariates were selected if deemed relevant to the outcome of interest and if questions were shown to both the PrEP-naive participants and current PrEP users. We used block entry to build the multivariable model using variables that were statistically significant at the bivariable level (at *p* < 0.05). We report unadjusted and adjusted odds ratios (OR and aOR) with 95% confidence intervals (CI). Statistical significance was set at *p* < 0.05 (two-tailed). Analyses were conducted using Stata version 16.0 (StataCorp, College Station, TX).

## Results

A total of 2046 surveys were completed in 2023. In this sample, 1907 (93.2%) participants were HIV-negative or untested. Of the 1907 non-HIV-positive participants, 33 (1.7%) had not heard of PrEP. Recently-arrived migrants were slightly less likely to be aware of PrEP (95.0%) than the other three groups (96.5%~99.0%, Fisher’s exact test, *p* = 0.015). For the main analysis, we included those who were willing to use PrEP but had never used it before (*n* = 228, hereafter referred to as the “willing non-users”) and current PrEP users (*n* = 947) (*n* = 1175, Table 1). Other participants (*n* = 732) were excluded from the analysis if they had never heard of PrEP, were not willing to use it, or had used it before but stopped. Most of the 1175 participants in the multivariable analysis completed the survey in English (*n* = 1129, 96.1%), followed by simplified Chinese (*n* = 30, 2.6%) and Thai (*n* = 16, 1.4%). The median age was 35 years old (interquartile range (IQR) 28–45) (Table 1). Of the 1175 participants, 1128 (96.0%) were cisgender men, 19 (1.6%) transgender men, and 28 (2.4%) non-binary people (Fisher’s exact test, *p* = 0.005). Among willing non-users (*n* = 228), most were cisgender men (*n* = 210, 92.1%); smaller proportions were transgender male (*n* = 8, 3.5%) or non-binary (*n* = 10, 4.4%). In relation to migration status, 28.3% of participants were born overseas, including 4.1% who had recently arrived (< 2 years). Among overseas-born participants (*n* = 333), Asia (41.7%) was the most commonly reported region of birth compared to Africa (5.1%), Latin America and the Caribbean (13.5%), Europe (9.6%), and high-income-English-speaking countries (30.0%; χ^2^(4, 333) = 9.83, *p* = 0.043). The top three reasons for migration were education (29.8%), for a job/career (29.5%) or for family/marriage/a relationship (25.3%), followed by another reason (13.0%) and tourism (2.4%; χ^2^(4, 333) = 14.06, *p* = 0.007). Most overseas-born participants migrated to Australia alone (58.9%) rather than with other people (41.1%; χ^2^(1, 333) = 0.43, *p* = 0.513). Most participants identified as gay (80.4%), lived in inner metropolitan areas (53.3%), had a university degree (67.1%), were employed full-time or part-time (88.1%), and had Medicare (Australia’s universal health insurance scheme) coverage (91.3%).

**Table 1:** Participant characteristics and multivariable logistic regression comparing PrEP users with willing non-users (*N* = 1175)

	Total	Current PrEP users	Willing non-users	Odds ratio	*p* Value	Adjusted odds ratio	*p* Value
	*N* = 1175	*n* = 947	*n* = 228				
Median age (IQR)	35.0 (28.0–45.0)	37.0 (29.0–47.0)	29.0 (23.0–38.0)	0.95 (0.93–0.96)	< 0.001*	0.97 (0.95–0.98)	< 0.001*
Migration status							
Australian-born	842 (71.7)	688 (72.7)	154 (67.5)	Ref.			
Born in high-income English-speaking countries	100 (8.5)	84 (8.9)	16 (7.0)	0.85(0.49–1.49)	0.574	0.97 (0.74–2.03)	0.942
Non-recently arrived migrants from other countries	185 (15.7)	151 (15.9)	34 (14.9)	1.01 (0.67–1.52)	0.977	0.93 (0.49–1.76)	0.823
Recently-arrived migrants from other countries	48 (4.1)	24 (2.5)	24 (10.5)	4.47 (2.47–8.08)	< 0.001*	5.04 (1.66–15.36)	0.004*
Sexual identity							
Gay	987 (84.0)	821 (86.7)	166 (72.8)	Ref.			
Bi/queer/other	188 (16.0)	126 (13.3)	62 (27.2)	2.43 (1.72–3.44)	< 0.001*	1.69 (1.02–2.80)	0.043*
State or territory							
New South Wales	386 (32.9)	313 (33.1)	73 (32.0)	Ref.			
Victoria	349 (29.7)	288 (30.4)	61 (26.8)	0.91 (0.62–1.32)	0.615		
Queensland	202 (17.2)	158 (16.7)	44 (19.3)	1.19 (0.78–1.82)	0.408		
Other states/territories	238 (20.3)	188 (19.9)	50 (21.9)	1.14 (0.76–1.71)	0.523		
Residential location^a^							
Inner metropolitan	626 (53.3)	529 (55.9)	97 (42.5)	Ref.			
Outer metropolitan	320 (27.2)	252 (26.6)	68 (29.8)	1.47 (1.04–2.08)	0.028*	1.26 (0.78–2.05)	0.346
Regional and remote	202 (17.2)	144 (15.2)	58 (25.4)	2.20 (1.51–3.19)	< 0.001*	1.76 (1.05–2.97)	0.033*
Education level							
High school	194 (16.5)	124 (13.1)	70 (30.7)	Ref.			
Trade certificate	193 (16.4)	158 (16.7)	35 (15.4)	0.39 (0.23–0.47)	< 0.001*	0.52 (0.26–1.02)	0.056
University degree	788 (67.1)	665 (70.2)	123 (53.9)	0.33 (0.23–0.47)	< 0.001*	0.65 (0.38–1.12)	0.120
Employment status							
Student/unemployed/other	140 (11.9)	94 (9.9)	46 (20.2)	Ref.			
Employed full-time or part time	1,035 (88.1)	853 (90.1)	182 (79.8)	0.44 (0.30–0.64)	< 0.001*	0.54 (0.30–0.97)	0.039*
Medicare coverage							
No	102 (8.7)	62 (6.5)	40 (17.5)	Ref.			
Yes	1,073 (91.3)	885 (93.5)	188 (82.5)	0.33 (0.21–0.50)	< 0.001*	1.68 (0.73–3.86)	0.22
No. of people known who are living with HIV							
None/don’t know	522 (44.4)	368 (38.9)	154 (67.5)	Ref.			
1–10 people	594 (50.6)	522 (55.1)	72 (31.6)	0.33 (0.24–0.45)	< 0.001*	0.81 (0.51–1.28)	0.358
10 + people	59 (5.0)	57 (6.0)	2 (0.9)	0.08 (0.02–3.35)	0.001*	0.38 (0.06–2.23)	0.285
No. of people known who are using PrEP							
None/don’t know	170 (14.5)	87 (9.2)	83 (36.4)	Ref.			
1–10 people	485 (41.3)	372 (39.3)	113 (49.6)	0.32 (0.22–0.56)	< 0.001*	0.60 (0.36-1.00)	0.051
10 + people	520 (44.3)	488 (51.5)	32 (14.0)	0.07 (0.04–0.11)	< 0.001*	0.34 (0.18–0.64)	0.001*
Self-rated likelihood of becoming HIV-positive							
Very unlikely/unlikely	1,026 (87.3)	858 (90.6)	168 (73.7)	Ref.			
Neither unlikely nor likely	125 (10.6)	79 (8.3)	46 (20.2)	2.97 (1.99–4.43)	< 0.001*	2.68 (1.53–4.68)	0.001*
Likely/Very likely	24 (2.0)	10 (1.1)	14 (6.1)	7.15 (3.12–16.37)	< 0.001*	8.74 (2.90-26.36)	< 0.001*
Any STI diagnosis (past 12mths)							
No	755 (64.3)	547 (57.8)	208 (91.2)	Ref.			
Yes	420 (35.7)	400 (42.2)	20 (8.8)	0.13 (0.08–0.21)	< 0.001*	0.41 (0.23–0.75)	0.003*
No. of male partners in last 6 months							
None	69 (5.9)	13 (1.4)	56 (24.6)	Ref.			
1–5	472 (40.2)	342 (36.1)	130 (57.0)	0.09 (0.05–0.17)	< 0.001*	0.29 (0.13–0.68)	0.004*
6–10	257 (21.9)	233 (24.6)	24 (10.5)	0.02 (0.01–0.05)	< 0.001*	0.22 (0.08–0.59)	0.003*
>10	377 (32.1)	359 (37.9)	18 (7.9)	0.01 (0.005–0.03)	< 0.001*	0.13 (0.05–0.38)	< 0.001*
Regular partner HIV status							
No regular partner(s)	708 (60.3)	565 (59.7)	143 (62.7)	Ref.			
HIV-negative	158 (13.4)	104 (11.0)	54 (23.7)	2.05 (1.40–2.99)	< 0.001*	2.24 (1.28–3.91)	0.005*
HIV-negative/Unknown, taking PrEP	237 (20.2)	229 (24.2)	8 (3.5)	0.14 (0.07–0.29)	< 0.001*	0.37 (0.16–0.86)	0.022*
Partner living with HIV	36 (3.1)	28 (3.0)	8 (3.5)	1.13 (0.50–2.53)	0.768	3.10 (1.00-9.63)	0.050
Unknown/untested	36 (3.1)	21 (2.2)	15 (6.6)	2.82 (1.42–5.61)	0.003*	1.74 (0.71–4.28)	0.229
Sex with regular male partners (past 6 months)							
No regular male partner(s) or no anal sex	322 (27.4)	201 (21.2)	121 (53.1)	Ref.			
Consistent condom use	42 (3.6)	26 (2.7)	16 (7.0)	1.02 (0.53–1.98)	0.948	2.04 (0.77–5.40)	0.152
Any condomless sex	811 (69.0)	720 (76.0)	91 (39.9)	0.21 (0.15–0.29)	< 0.001*	0.85 (0.52–1.40)	0.530
Sex with casual male partners (past 6 months)							
No casual male partner(s) or no anal sex	243 (20.7)	103 (10.9)	140 (61.4)	Ref.			
Consistent condom use	84 (7.1)	56 (5.9)	28 (12.3)	0.37 (0.22–0.62)	< 0.001*	0.33 (0.16–0.69)	0.003*
Any condomless sex	848 (72.2)	788 (83.2)	60 (26.3)	0.06 (0.04–0.08)	< 0.001*	0.17 (0.10–0.29)_	< 0.001*
Any party drug use (past 6 months)							
No	965 (82.1)	758 (80.0)	207 (90.8)	Ref.			
Yes	210 (17.9)	189 (20.0)	21 (9.2)	0.41 (0.25–0.66)	< 0.001*	0.86 (0.44–1.65)	0.639

^a^The *Residential location* variable was classified based on the postcode provided by participants. Some individuals did not provide a postcode and were thus categorized as No response (*n* = 22) for *Residential location*. To maintain data integrity and simplify the analysis, this category was removed from the table. As a result, the total number of participants in the remaining categories (*Inner metro*, *Outer metro*, and *Regional and remote*, *n = 1153*) is slightly lower than the overall sample size (*N* = 1175). In the multivariable logistic regression analysis, with *Inner metro* as the reference category, the removal of No response did not alter the overall direction of the results. *p<0.05

Table 1 presents the characteristics of participants who were willing to use PrEP but not currently using it, compared with current PrEP users, and the multivariable regression results. Compared with current PrEP users, willing non-users were younger, more likely to be recently-arrived migrants, more likely to identify as bisexual/queer/other compared to gay, and more likely to live in regional and remote areas compared to inner-metropolitan areas, but less likely to be employed compared to any other non-employed category (student, unemployed, or other). Willing non-users were less likely to know other PrEP users than current PrEP users. Willing non-users were more likely to perceive themselves as being likely/very likely to acquire HIV, but less likely to report any STI diagnosis in the last 12 months. Willing non-users had fewer male partners, compared with current PrEP users. Willing non-users were more likely to have a HIV-negative regular partner who was not on PrEP, and less likely than PrEP users to have a regular partner who was taking PrEP. Compared with current PrEP users, willing non-users had lower odds of engaging in anal sex with casual partners, whether condoms were used.

## Discussion

We assessed differences in PrEP use by country of birth and migration status among GBM and non-binary people in Australia. We found that recently-arrived migrants were much less likely than Australian-born participants and other migrants to report current PrEP use, highlighting the need for easier access to PrEP for recently-arrived migrants in Australia. We then assessed factors associated with being willing to use PrEP but not currently using it and found that recently arrived migrants from non-high-income-English-speaking countries were also overrepresented in the group who wanted to use PrEP. Other factors associated with willingness to use PrEP among non-users included younger age, being non-gay-identified, and not being employed (e.g., students, unemployed people and those receiving government benefits), highlighting groups in addition to recently-arrived migrants in need of easier access to PrEP. While PrEP uptake has increased substantially in recent years in Australia [1, 5, 13], our findings underscore that this success has not been equitably experienced across all populations, aligning with previous research [2, 9, 15]. Building on existing evidence about the factors influencing current PrEP use, our findings highlight the need to strengthen the focus on migrant populations, particularly those who have recently arrived, to promote equitable access and reduce disparities in HIV prevention in Australia.

Consistent with previous studies, we found people not using PrEP but willing to use it were more likely to be younger, unemployed, and living outside of inner-city areas [13, 26–28]. These socioeconomic and structural factors may limit their access to healthcare, including the cost of the medication, attending clinical appointments, finding accessible and LGBTQ+ friendly services for HIV and STI screening, and obtaining new prescriptions [15, 17]. Additionally, we found willing non-users were less sexually active, and less socially engaged with other PrEP users. Compared with current PrEP users, willing non-users had fewer sexual partners, lower rates of condomless sex, fewer STI diagnoses, lower levels of illicit drug use, and fewer connections with PrEP users, aligning with previous research [12, 13, 29]. This highlights a challenge in supporting risk-informed decision-making about PrEP among less sexually active GBM and non-binary people if they do not consider themselves to be at sufficient risk of HIV to use PrEP (and if they have concerns about whether they can afford to access and use it). A solution may be to highlight on-demand or event-driven dosing (taking oral PrEP shortly before and for a few days after sex), noting that this dosing method is as effective as daily dosing if practiced accurately, and can be seen as more appropriate by people who are less sexually active [30]. Our analysis of migration status in relation to PrEP awareness, willingness to use PrEP, and current PrEP use aligns with and strengthens the findings of previous studies. For example, in previous research recently-arrived GBM and non-binary people, including international students, reported lower levels of PrEP awareness compared to Australian-born people [3, 31]. Another Australian study found that many migrant GBM and non-binary people were willing to use PrEP but their level of uptake remained low [18]. In both Australian and international contexts, the financial burden associated with PrEP use, including costs for appointments and tests, is recognised as a barrier for migrants without access to subsidised health care (e.g., Medicare in Australia) or private health insurance [13, 15, 32–34]. Australian research has shown that providing PrEP for free with translated information can significantly improve PrEP adherence and uptake by migrants without Medicare access [33]. Free or low-cost access schemes, supported by translators, publicised through educational institutions and PrEP users’ social networks, may help promote PrEP uptake among recently arrived GBM [13, 33, 34].

Several variables were significantly associated with PrEP use at a bivariable level but not independently associated with PrEP in the multivariable model, when controlling for the other variables. These included Medicare coverage, education level, knowing people living with HIV, having condomless sex with regular partners, and party drug use. In most cases, we believe these variables became nonsignificant in the multivariable model because they were related to other variables in the model which had stronger relationships with PrEP use. For example, Medicare coverage is primarily affected by migration status (with longer term residents much more likely to have Medicare coverage than recently-arrived migrants). For the other variables, the final model contained other indicators of capacity to access PrEP, and markers of potential HIV risk, that may be more directly related to PrEP use, such as being in paid employment, having a recent STI diagnosis, and the number of recent sex partners.

We acknowledge that there are limitations to our study. We recruited a convenience sample that may not be representative of GBM nationally, potentially leading to an overrepresentation of individuals who were more interested in PrEP. While our survey offered language options in English, Chinese, and Thai, this may have limited participation from individuals who use other languages or those less proficient in English, potentially affecting the diversity of our sample. However, as our data were collected through community-based recruitment, this approach may have enhanced the accessibility of the study to diverse groups within the community. Additionally, our findings rely on self-reported data, which may be subject to recall or social desirability biases. However, our survey was anonymous and self-administered online, which may have encouraged more honest reporting of personal information. Migration status in this study was operationalised using country of birth and length of residence in Australia, following previous research [3, 9, 24]. While this classification facilitates comparisons across studies, it simplifies substantial heterogeneity within and between participants’ countries of origin. Importantly, differences in the availability of and access to health systems and HIV prevention may vary not only between countries, but also within participants’ countries or regions of origin. As a result, categorising participants by country of birth may mask meaningful variations in health access and literacy, prior exposure to HIV prevention, and familiarity with health services. Future research would benefit from more granular measures of participant backgrounds that may affect access to HIV services, such as region of origin within a participant’s country of birth, citizenship or residency status, race or ethnicity, and prior access to HIV-related health services.

## Conclusion

This study highlights the uneven distribution of PrEP uptake among GBM and non-binary people in Australia, with PrEP use significantly lower among recently-arrived migrants. The findings underscore the need for targeted interventions to address barriers to PrEP use faced by recently-arrived migrants, younger people, and people in regional areas. To promote equitable PrEP uptake and contribute to improved HIV prevention coverage, we recommend greater use of free or low-cost PrEP access schemes, supported by multilingual services, using educational institutions and peer networks for outreach and information sharing.

## Data Availability

The data that support this study cannot be publicly shared due to ethical and privacy considerations. However, the syntax used for database coding and analysis may be made available upon reasonable request to the corresponding author.
